# Insight into the Characteristics of Novel Desmin-Immunopositive Perivascular Cells of the Anterior Pituitary Gland Using Transmission and Focused Ion Beam Scanning Electron Microscopy

**DOI:** 10.3390/ijms22168630

**Published:** 2021-08-11

**Authors:** Depicha Jindatip, Rebecca Wan-Yan Poh, Ken Fujiwara

**Affiliations:** 1 Department of Anatomy, Faculty of Medicine, Chulalongkorn University, 1873 Rama 4 Rd., Wangmai, Pathumwan, Bangkok 10330, Thailand; 2 Division of Histology and Cell Biology, Department of Anatomy, Jichi Medical University, School of Medicine, 3311-1 Yakushiji, Shimotsuke 329-0498, Japan; fujiwarak@kanagawa-u.ac.jp; 3 Microscopy Business Group, Carl Zeiss Pte. Ltd., 50 Kaki Bukit Place, 05-01, Singapore 415926, Singapore; rebecca.poh@thermofisher.com; 4 Department of Biological Sciences, Faculty of Science, Shonan Hiratsuka Campus, Kanagawa University, 2946 Tsuchiya Hiratsuka, Hiratsuka 259-1293, Japan

**Keywords:** capsular fibroblast, desmin, dilated cisternae of rough endoplasmic reticulum, folliculostellate cell, macrophage, pericyte, three-dimensional reconstruction

## Abstract

Recently, another new cell type was found in the perivascular space called a novel desmin-immunopositive perivascular (DIP) cell. However, the differences between this novel cell type and other nonhormone-producing cells have not been clarified. Therefore, we introduced several microscopic techniques to gain insight into the morphological characteristics of this novel DIP cell. We succeeded in identifying novel DIP cells under light microscopy using desmin immunocryosection, combining resin embedding blocks and immunoelectron microscopy. In conventional transmission electron microscopy, folliculostellate cells, capsular fibroblasts, macrophages, and pericytes presented a flat cisternae of rough endoplasmic reticulum, whereas those of novel DIP cells had a dilated pattern. The number of novel DIP cells was greatest in the intact rats, though nearly disappeared under prolactinoma conditions. Additionally, focused ion beam scanning electron microscopy showed that these novel DIP cells had multidirectional processes and some processes reached the capillary, but these processes did not tightly wrap the vessel, as is the case with pericytes. Interestingly, we found that the rough endoplasmic reticulum was globular and dispersed throughout the cytoplasmic processes after three-dimensional reconstruction. This study clearly confirms that novel DIP cells are a new cell type in the rat anterior pituitary gland, with unique characteristics.

## 1. Introduction

Five mixed types of hormone-producing cells in the anterior pituitary gland assemble in clumps and cords, forming cluster structures [1,2]. Generally, each cell group is surrounded by a parenchymal basement membrane that separates hormone-cell clusters from the perivascular space and capillaries. In addition to hormone-producing cells, there are other cell types that do not produce hormones in the gland, i.e., folliculostellate cells, capsular fibroblasts, macrophages, endothelial cells, and pericytes. In 2012, we succeeded in identifying a new cell type in the perivascular space using desmin immunoelectron microscopy; namely, novel desmin-immunopositive perivascular (DIP) cells [3]. However, the differences in morphology and characteristics between this novel DIP cell and other nonhormone-producing cells have not been compared and elucidated.

Focused ion beam scanning electron microscopy (FIB-SEM) is a new developmental scanning electron microscope that contains dual beams to mill a block face and scan surface specimens. A serial stack of hundreds of transmission electron-like microphotographs is obtained after repeating the “milling and scanning” procedure. Tomography of the entire cell structure can be carried out to create a reconstruction using three-dimensional (3D) visualization. Currently, the application of FIB-SEM with 3D analysis has become a powerful tool for biological research [4,5]. This technique is widely used to analyze the synaptic area of neurons [6,7,8]. Di Giulio and Muzzi (2018) mentioned the usefulness of the FIB-SEM procedure to study the hard exoskeleton of arthropods instead of using ultramicrotome sectioning and conventional transmission electron microscopic observation [9]. In the anterior pituitary gland, Yoshitomi et al. (2016) applied FIB-SEM tomography alongside immunostaining techniques to classify five types of endocrine cell, and showed that the different patterns of secretory granule distribution depended on the relationship between endocrine cell location and capillaries [10]. In the present study, we performed desmin immunoelectron and conventional transmission electron microscopy to reveal the characteristics of novel DIP cells compared to other cells in the gland. Moreover, FIB-SEM could potentially clarify the 3D morphology of the novel DIP cells. Findings from this study could provide a better understanding of fine structures and the entire external cell shape of the novel DIP cells, including physical interactions between the novel DIP cells and other cell populations in the rat anterior pituitary gland.

## 2. Results

### 2.1. Light Microscopy

**Figure 1:**
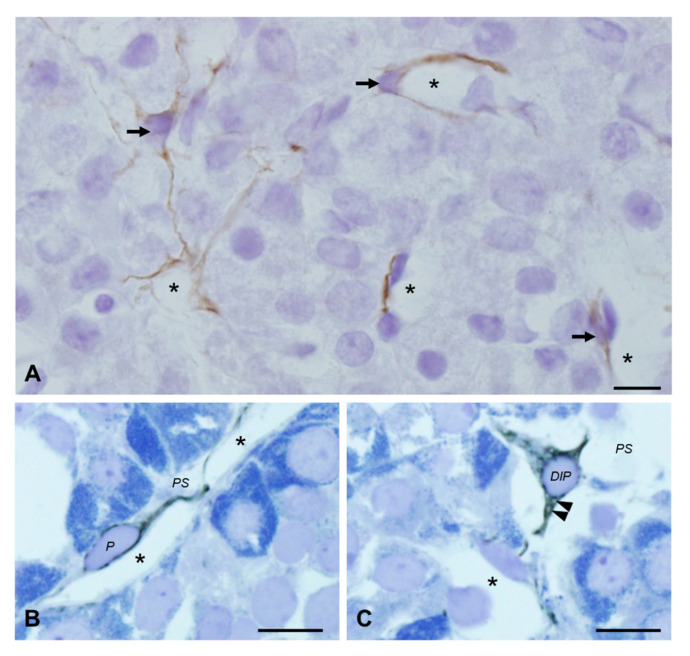
Light microscopy of desmin immunohistochemistry in the anterior pituitary glands of adult normal rats (**A**–**C**). Homogeneous brown immunopositive signals (arrows) appear on the typical 8-µm cryosections representing both pericytes and novel desmin-immunopositive perivascular cells (**A**), whereas semithin sections of immunocryo-epoxy resin embedding blocks with toluidine blue counterstaining reveal morphological differences between pericytes (*P* in **B**) and novel desmin-immunopositive perivascular cells (*DIP* in **C**). Unstained circular cavities in the black cytoplasm of novel desmin-immunopositive perivascular cells (arrowheads) are the dilated lumen of rERs: capillaries (asterisks); perivascular space (*PS*). Bars 10 µm.

Cryosections of 8 µm in thickness and stained by desmin immunohistochemistry with hematoxylin counterstaining were observed first. All brown positive signals were visible in the cytoplasm and cytoplasmic processes of perivascular cells, which presented characteristics of pericytes and novel DIP cells. However, we could not distinguish novel DIP cells from pericytes using this technique, as it showed a homogeneous staining pattern (Figure 1A).

Consequently, semithin resin sections with toluidine blue were used to analyze the desmin-stained cells instead. The immunopositive cells were black, as shown in Figure 1B,C. Pericytes had slim cytoplasm and thin processes with intense black staining, which were found to be close to the capillary (Figure 1B). Notably, this semithin resin could clearly identify novel DIP cells by revealing small unstained circular cavities in their cell cytoplasm at the light microscopic level (Figure 1C). Moreover, the black staining of desmin did not fully disperse throughout the entire cytoplasm and processes of novel DIP cells, as is the case with pericytes (Figure 1B,C, Supplemental Figure S1).

### 2.2. Immunoelectron Microscopy

**Figure 2:**
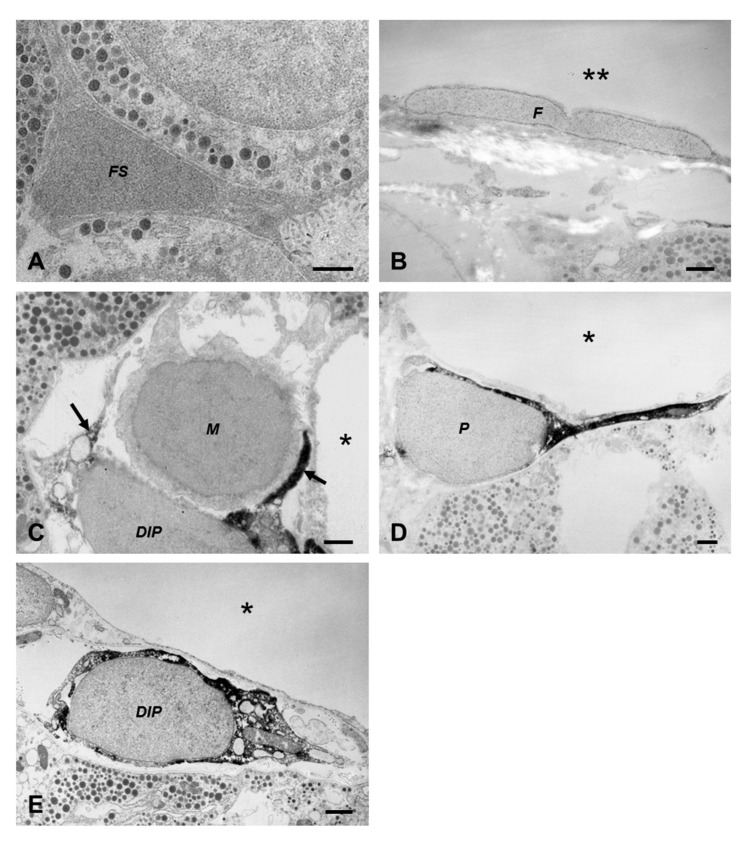
Desmin immunoelectron micrographs of nonhormone-producing cells in the rat anterior pituitary gland (**A**–**E**). A black positive signal of desmin is visible in pericytes and novel desmin-immunopositive perivascular cells, confirming the results from light microscopy: folliculostellate cell (*FS* in **A**); fibroblast at pituitary capsule (*F* in **B**); macrophage (*M* in **C**); pericyte (*P* in **D**); novel desmin-immunopositive perivascular cell (*DIP* in **C**,**E**); area outside the gland (double asterisks in **B**); capillaries (asterisks in **C**–**E**); cytoplasmic process of adjacent novel desmin-immunopositive perivascular cell (arrow in **C**). Bars 1 µm.

**Figure 3:**
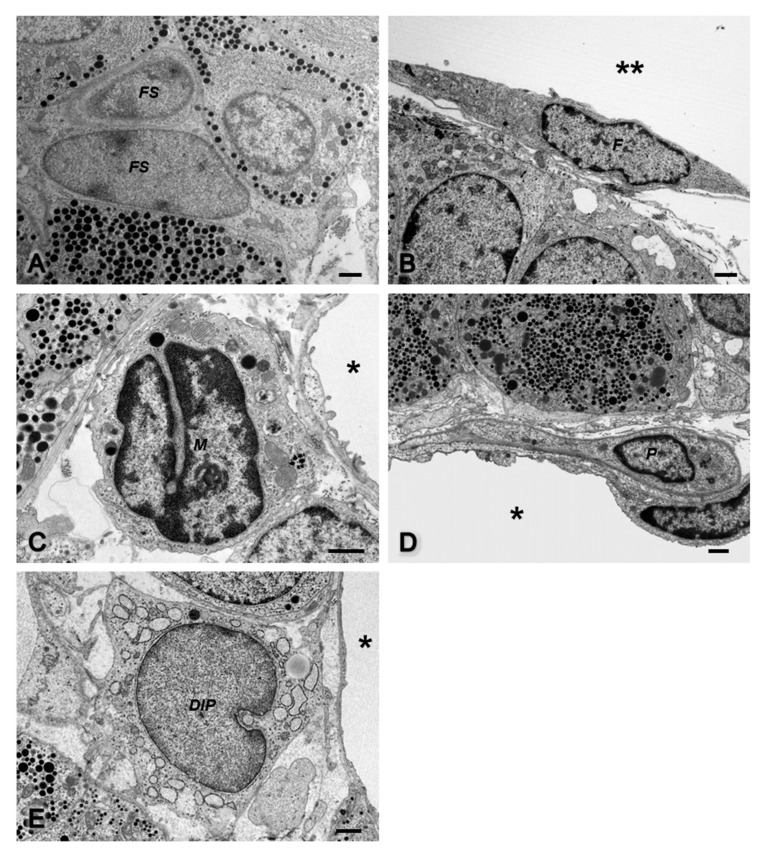
Conventional transmission electron microscopy of nonhormone-producing cells in the rat anterior pituitary gland (**A**–**E**): folliculostellate cells (*FS* in **A**); fibroblast at pituitary capsule (*F* in **B**); macrophage (*M* in **C**); pericyte (*P* in **D**); novel desmin-immunopositive perivascular cell (*DIP* in **E**); area outside the gland (double asterisks in **B**); capillaries (asterisks in **C**–**E**). Bars 1 µm.

To confirm the results from the light microscopy, immunoelectron microscopy of desmin was carried out. The immunoreaction was negative in folliculostellate cells (Figure 2A), capsular fibroblasts (Figure 2B), macrophages (Figure 2C), and all hormone-producing cell types (data not shown), whereas this antibody was restrictively observed in pericytes (Figure 2D) and novel DIP cells (Figure 2E). Figure 2E demonstrates that some novel DIP cells were also localized near the capillary lumen in the narrow perivascular space as pericytes. Noticeably, macrophages and novel DIP cells (arrows in Figure 2C) were often located together. Combined with the immunoelectron microscopic study of desmin, conventional ultrathin sections were observed to obtain details of the fine structure of each cell type (Figure 3).

### 2.3. Conventional Transmission Electron Microscopy

Folliculostellate cells (Figure 3A) were found within the parenchymal basement membrane and usually formed a cluster in the midst of the hormone cells. Fibroblasts (Figure 3B) of the rat anterior pituitary gland were localized in the outermost capsular connective tissue. These capsular fibroblasts were fusiform in shape, and their cell organelles showed an active state. Interestingly, there were no cells that exhibited fibroblast characteristics inside the gland.

In the perivascular space, three types of nonhormone-producing cells occupied this area, i.e., macrophages, pericytes, and novel DIP cells. Macrophages (Figure 3C) had irregular shapes with a large cytoplasm containing many lysosomes and engulfed vacuoles. The number of macrophages in this normal condition was lower. Pericytes (Figure 3D) were visible underneath the vascular basement membrane shared with the endothelial cells and had fine processes that surrounded the capillary. When the anterior pituitary was under normal conditions, pericytes were quiescent, with a small cytoplasmic volume, and their cell bodies were in close contact with the endothelial cells. Notably, all cells described above had narrow lumens of the rough endoplasmic reticulum (rER) network, whereas the rER of the novel DIP cells had an expanded lumen appearance (Figure 3E). Our pilot study found that this novel cell type was detected in the anterior pituitary gland of several breeds, such as Wistar, Sprague Dawley, LEXF RI rats, and BALB/c nude mice with identical morphology (data not shown). They had no basement membrane and showed a number of dilated cisternae of rERs in the expanded cytoplasm. Lysosomes and lipid droplets were visible. 

**Figure 4:**
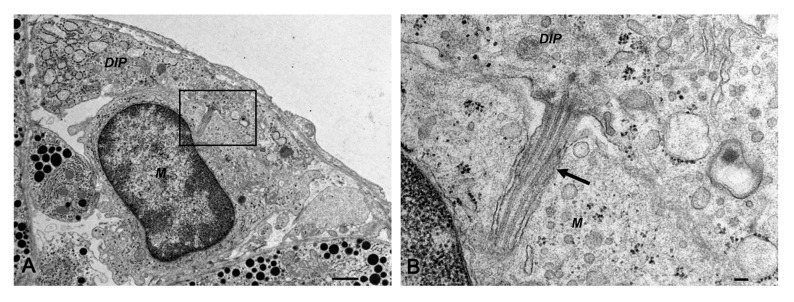
Conventional transmission electron microscopy of novel desmin-immunopositive perivascular cells, *DIP* and macrophages, *M* (**A**,**B**: higher magnification of squared area in **A**). A single cilium (arrow) extends from the novel desmin-immunopositive perivascular cell type. Bars 1 µm (**A**), 0.1 µm (**B**).

Novel DIP cells did not make direct contact with pericytes because they were separated by the vascular basement membrane. However, we noticed that novel DIP cells physically came into contact with macrophages (11% of 181 novel DIP cells counted from three rats), and we sometimes found a single cilium extending from the cytoplasm of novel DIP cells that were inserted into the adjacent macrophages, as shown in Figure 4. The rest were isolated novel DIP cells that did not make contact with macrophages (89%). It was noted that the novel DIP cells were not found in the posterior and intermediate pituitary lobes.

**Figure 5:**
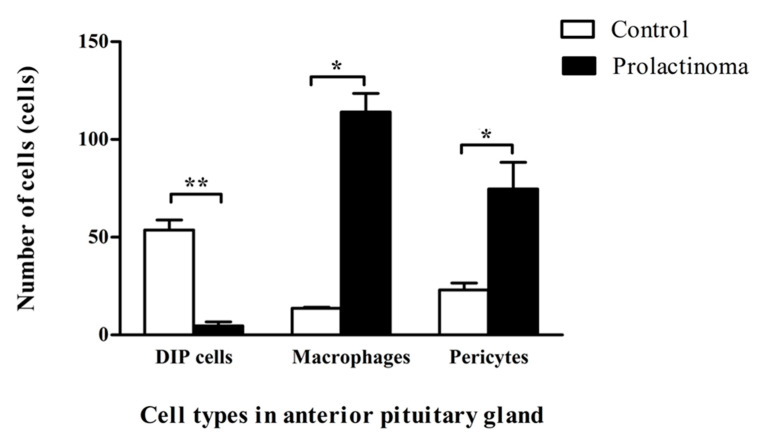
Number of cells in the perivascular space of anterior pituitary glands in control (*n* = 3 rats) and prolactinoma rats (*n* = 3 rats). Values are the mean ±  SEM. Desmin-immunopositive perivascular (DIP) cells, ** *p*  <  0.001; macrophages, * *p*  <  0.005; and pericytes, * *p*  <  0.005.

Additionally, three types of perivascular cell in LEXF RI rats were counted to compare intact rats and rats with pathological conditions—a diethylstilbestrol (DES)-induced prolactinoma rat model. We used ultrathin sections for this experiment because all types of perivascular cells could be clearly identified using transmission electron microscopy (Supplemental Figure S2). Three mid-sagittal pituitary glands were selected per group, and each specimen was defined as a 6 × 6 square on a 150-mesh copper grid to represent the area of cell counting. One square of the grid is approximately 116 µm × 118 µm (Supplemental Figure S3). In the control rats, a number of novel DIP cells were observed (53.67 ± 5.13), followed by pericytes (23.00 ± 3.60) and macrophages (13.67 ± 0.58). Surprisingly, the number of novel DIP cells was significantly decreased in DES-induced prolactinoma rats (4.67 ± 2.08), whereas the number of pericytes and macrophages were significantly increased (74.67 ± 13.65 and 114.00 ± 9.54, respectively) compared to the untreated control rats (Figure 5).

### 2.4. FIB-SEM and 3D Reconstruction of the Novel Desmin-Immunopositive Perivascular Cell

**Figure 6:**
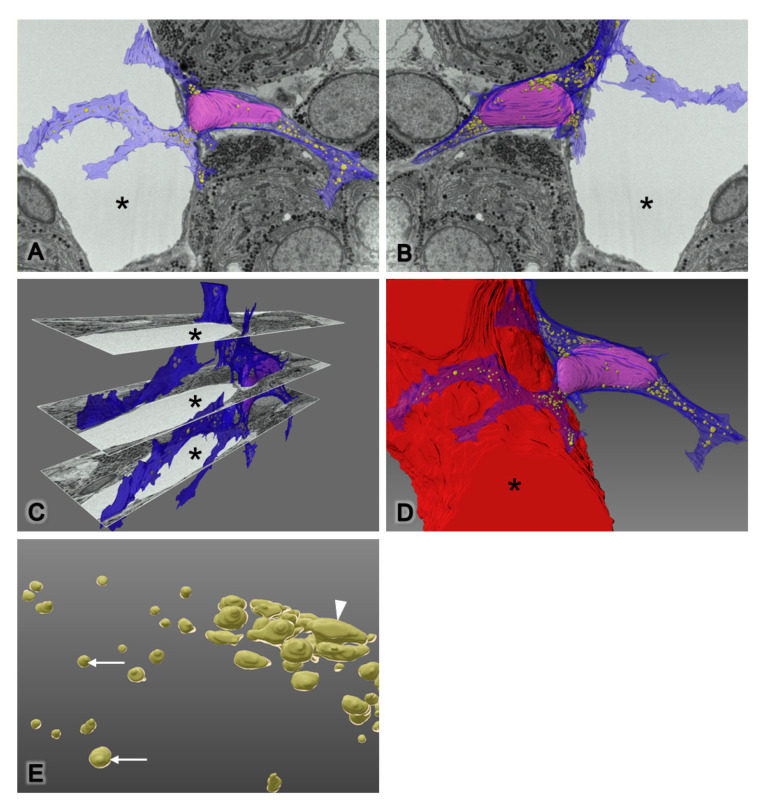
Three-dimensional reconstruction of novel desmin-immunopositive perivascular cells: anterior (**A**), posterior (**B**), and luminal diagonal views (**C**). Three-dimensional diagram shows the relationship between DIP cell and capillary (**D**). The globular and anastomotic rER appearances are white arrows and white arrowhead, respectively (**E**). Capillaries are asterisks, pseudocolor of cytoplasm is blue, pseudocolor of nucleus is pink, and pseudocolor of rER bodies are yellow, magnification is 2800×, image pixel size on the XY plane is 19.937 nm, and resolution on the Z plane is 60 nm.

To visualize the 3D morphology of the novel DIP cells more accurately, serial images of the whole cell were obtained from FIB-SEM. These datasets of 2D serial images had inverted contrast, making them appear similar to conventional transmission electron micrographs. The image stack revealed various features of the cell shape of novel DIP cells at each level until the entire cell was complete (Supplemental Figure S4A–F). The novel DIP cells had a long and expanded cytoplasm with multidirectional projections. Some of their processes reached the parenchymal interstitium and some reached the capillary wall. Additionally, FIB-SEM tomographs confirmed that a group of microtubule protrusions (Supplemental Figure S4D) was a single primary cilium. This cilium extended from the mother centriole of the centrosome (Supplemental Figure S5A–C) and was orthogonally arranged with the daughter centriole (Supplemental Figure S5C,D). For the 3D reconstruction, approximately 200–250 serial images were used to create the whole shape of the novel DIP cells, including cell processes. All directions of the cell are shown in Figure 6 and Supplemental Video S1. Dilated cisternae of rERs were globular and short tubular in shape (Supplemental Figure S6), with various sizes. A number of these rER bodies are scattered in the cytoplasmic processes. Anastomosis of the globular and short tubular rER cisternae forming the ER network could be seen around the nuclear area (Figure 6E). 

## 3. Discussion

Desmin immunohistochemistry and immunoelectron microscopy showed that all hormone-producing cells, folliculostellate cells, macrophages, capsular fibroblasts, and endothelial cells were negatively stained, except novel DIP cells and pericytes. Conventional transmission electron microscopy revealed the differences between novel DIP cells and other cell populations in the gland. Moreover, focused ion beam scanning electron microscopy displayed the precise cell shape and globular pattern of the rough endoplasmic reticulum of the novel DIP cell.

A novel DIP cell is a new cell type localized in the anterior pituitary gland and is not found in the intermediate or posterior lobe. Our previous studies reported that this cell type was observed in normal rats from early postnatal development until adulthood, and was definitively identified by transmission electron microscopy [3,11]. In the present study, we analyzed the novel DIP cells under light microscopy using desmin immunocryosection with pre-embedded resin sections (Figure 1). The osmium tetroxide binding DAB enhanced the contrast between the stained and unstained areas in the cell cytoplasm, which was suitable for identifying our new cell type specifically. Desmin immunoelectron microscopy clearly demonstrated that there were some novel DIP cells adjacent to the capillary as pericytes. However, both cells presented different morphologies, as shown in Figure 2. Indeed, the expression of desmin in fibroblasts is still controversial. Several publications have shown that fibroblasts do not express positive desmin immunohistochemistry signals [12,13,14]. Therefore, we assumed that the fibroblasts of the rat anterior pituitary were also desmin-negative cells, and were specifically found in the capsular connective tissue, wrapping around the entire gland. In addition, the cell shape and nucleus of these capsular fibroblasts were fusiform (Figure 3), resembling fibroblasts in other organs [15,16], and we did not observe this characteristic in the perivascular space inside the anterior pituitary lobe. Moreover, we have demonstrated that novel DIP cells and pericytes play a role as collagen-expressing cells in the rat anterior pituitary gland instead of fibroblasts [11,17,18], though they need to interact with FS cells for collagen synthesis [19]. 

In the perivascular space of intact adult rats, novel DIP cells are a major cell type. They were occasionally found with macrophages and could interact with each other via axoneme insertion (Figure 2C and Figure 4). This structure is a single cilium of the centrosome which is involved in cell-to-cell signaling and communication [20]. Interestingly, prolactinoma had a significant negative effect on the number of novel DIP cells (Figure 5). Therefore, the interaction between these perivascular cells might be lost due to the absence of novel DIP cells. However, an imbalance in the cell populations in response to pathological inflammation needs to be demonstrated in further studies.

In the last decade, the 3D reconstruction of tomography imaging has been useful to clarify the accurate morphology of fine structures inside cells and has tended to increase the number of publications in this field [10,21,22,23,24]. We introduced FIB-SEM, a new scanning electron microscopic technique, to investigate the entire cell shape, including organelles, of novel DIP cells. Instead of conventional manual ultramicrotome sectioning, FIB-SEM rapidly provides stacks of transmission electron-like micrographs using an automatic milling-scanning system. The FIB-SEM system works with the same resin blocks as a conventional transmission electron microscopy and is also able to apply a metal coating on the surface of the blocks. Therefore, it is useful for histologists who would like to enter the 3D biological field. In this study, tomography showed the differences in cell shapes when observing the image stack, and we could not gain these data from a single capture of 2D conventional transmission electron micrographs. Yoshitomi et al. (2016) reported that a number of pituitary hormone cells were arranged close to the vessel wall to facilitate hormone secretion [10]. This finding supports our hypothesis that novel DIP cells might communicate or associate with capillaries, as they also reached the processes and wrapped around the capillary. Moreover, our study is the first report of the ball-like structure and short anastomosis of rERs in vivo using 3D reconstruction technology (Figure 6 and Supplemental Video S1).

## 4. Materials and Methods

### 4.1. Animals

Eight-week-old male Wistar rats were obtained from the National Laboratory Animal Center (Nakhon Pathom, Thailand). These animals were given food and water ad libitum and maintained under a 12:12 h light/dark cycle at a controlled temperature of approximately 22 °C. All procedures were performed in accordance with the Guidelines for Animal Experimentation of the Faculty of Medicine, Chulalongkorn University, which are based on the guidelines of the National Research Council of Thailand.

In addition, resin blocks of the pathological prolactinoma model were obtained from our previous study [25]. Protocol of the diethylstilbestrol (DES)-induced prolactinomas was briefly described, as follows: LEXF RI rats (LE/Stm  ×  F344) subcutaneously implanted with a silastic tube (inner diameter: 2 mm, outer diameter: 3 mm, length: 20 mm; Kaneka Medix, Osaka, Japan) containing diethylstilbestrol (Sigma-Aldrich, St. Louis, MO, USA) for 3 months.

### 4.2. Light Microscopy

Desmin immunohistochemistry was introduced to identify novel DIP cells, including pericytes in the anterior pituitary. Rats were sacrificed using isoflurane and then transcardially perfused with 4% paraformaldehyde (Cat. no. 818715, Merck Millipore, Darmstadt, Germany) in 0.05 M phosphate buffer (PB, pH = 7.4) for 24 h at 4 °C. Pituitary glands were continually immersed in the same fixative, and then in 30% sucrose in 0.05 M PB (pH = 7.2) for two days. The glands were embedded in Tissue-Tek O.C.T. Compound (Cat. No. 4583, Sakura Finetechnical, Tokyo, Japan), frozen, and cut into 8 µm and 50 µm thick sections by a cryostat (CM1950; Leica Microsystems, Wetzlar, Germany). Both 8 µm and 50 µm cryosections were treated with 2% normal goat serum (Cat. no. S-1000, Vector Laboratories, Burlingame, CA, USA) in phosphate-buffered saline (PBS) for 20 min at 30 °C and with anti-human desmin rabbit polyclonal antibody (diluted 1:1500, Cat. no. ab15200, Abcam, Cambridge, UK) in PBS overnight, at room temperature. Then, the sections were incubated by biotinylated anti-rabbit IgG (Cat. no. BA-1000, Vector Laboratories, Burlingame, CA, USA) for 30 min at 30 °C, and the ABC method (Cat. no. PK-4000, Vector Laboratories, Burlingame, CA, USA) with 3,3′-diaminobenzidine (Cat. no. D5637, Sigma-Aldrich, St. Louis, MO, USA). After finishing the process of desmin immunohistochemistry, 8 µm cryosections were observed under a light microscope (AX80; Olympus, Tokyo, Japan), while 50 µm thick cryosections were continually performed with 1% osmium tetroxide (OsO_4_, Cat. no. 19110, EMS, Hatfield, PA, USA) for 90 min, followed by epoxy resin embedding and 1.5 µm semithin sectioning with an ultramicrotome (EM UC7, Leica, Vienna, Austria). These semithin sections were stained with toluidine blue, analyzed by light microscopy, and then compared with the 8 µm cryosections.

### 4.3. Immunoelectron and Conventional Transmission Electron Microscopy

After observing semithin sections at the light microscopic level, desmin immunocryo resin blocks were continually cut at 70 nm in thickness to make ultrathin sections. Positive signals of desmin on the tissue were visualized by transmission electron microscopy (JEM-1400PLUS; JEOL, Tokyo, Japan). In addition, the fine structure of nonhormone-producing cells, i.e., fibroblasts, folliculostellate cells, macrophages, pericytes, and novel DIP cells, was demonstrated by conventional transmission electron microscopy. In brief, three animals were perfused with 2% glutaraldehyde (Cat. no. 16220, EMS, Hatfield, PA, USA) in 0.05 M PB (pH 7.4) for 5 min, and pituitary glands were removed. Each gland was sagittally sliced into small sections, and immediately immersed in the same fixative for 2 h. Then, these pituitary pieces were treated with 1% OsO_4_, dehydrated by alcohol series (Cat. no. 1000983, Merck Millipore, Darmstadt, Germany) and propylene oxide (Cat. no. 807027, Merck Millipore, Darmstadt, Germany), and embedded in epoxy resin. After cutting blocks to 70 nm in thickness, ultrathin sections were stained with lead citrate (Cat. no. 178000, EMS, Hatfield, PA, USA) and uranyl acetate (Cat. no. 22400, EMS, Hatfield, PA, USA). 

### 4.4. Focused Ion Beam Scanning Electron Microscopy 

Three pituitary glands were prepared in resin blocks using the same steps for conventional electron microscopy described above. The specimen surface was coated with an ion sputter coater (Emitech SC7620; Quarum Technologies, Kent, UK). Then, the specimens were inserted into the vacuum chamber of the focused ion beam scanning electron microscope (FIB-SEM; Auriga 60; Carl Zeiss Microscopy, Oberkochen, Germany) and tilted at 54° to a gallium ion beam. The selected area on the resin block face was milled away by 60 nm, using ion beams of 30 kV and 1 nA, and imaged by a scanning electron microscopic system with a back-scattered electron detector (Supplemental Figure S7). The FIB-SEM process was repeated step by step until the entire cell was completed. Stacks of transmission electron microscopy-like images of novel DIP cells were obtained, and 3D reconstruction was created using Imaris software version 9.3.2 (Bitplane, Belfast, UK).

## 5. Conclusions

Our present study applies several microscopic techniques, including advanced microscopic technology and FIB-SEM tomography, to create a 3D reconstruction of novel desmin-immunopositive perivascular cells in rat anterior pituitary glands. Future directions for this research include quantifying the number, diameter, and 3D volume of dilated rERs in the novel DIP cells, as well as identifying this novel cell type and investigating its function in the human anterior pituitary gland.

## Data Availability

Data sharing is not applicable to this article.

## Supplementary Materials

The following are available online at https://www.mdpi.com/article/10.3390/ijms22168630/s1.
