# Supplementary material for: Insight into the Characteristics of Novel Desmin-Immunopositive Perivascular Cells of the Anterior Pituitary Gland Using Transmission and Focused Ion Beam Scanning Electron Microscopy
Source: Int J Mol Sci. 2021 Aug 11;22(16):8630. doi: 10.3390/ijms22168630 (PMC8395444; doi:10.3390/ijms22168630)
Supplement: Supplementary file 1 [file ijms-22-08630-s001.zip › Supplementary Information Jindatip et al 2021.pdf]

Supplementary Information:

Insight into the characteristics of novel desmin-immunopositive perivascular cells of the anterior pituitary gland using transmission and focused ion beam scanning electron microscopy

Depicha Jindatip, Rebecca Wan-Yan Poh and Ken Fujiwara

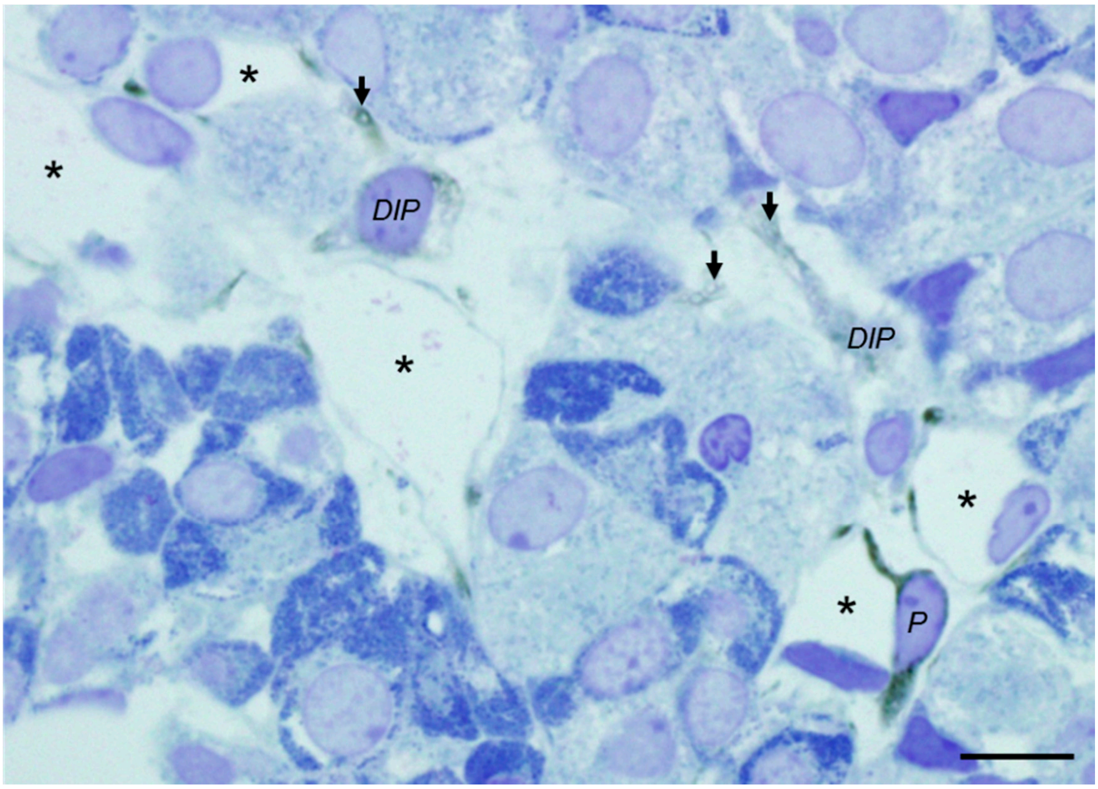

Figure S1. Comparison of the desmin immunostaining pattern between pericytes (*P*) and novel desmin-immunopositive perivascular cells (*DIP*) in the same area on an immunocryo-epoxy resin embedding block. Note capillaries (asterisks), cytoplasmic process segments of novel desmin-immunopositive perivascular cells with low intensity of immunoreaction (arrows). Bar 10 μm

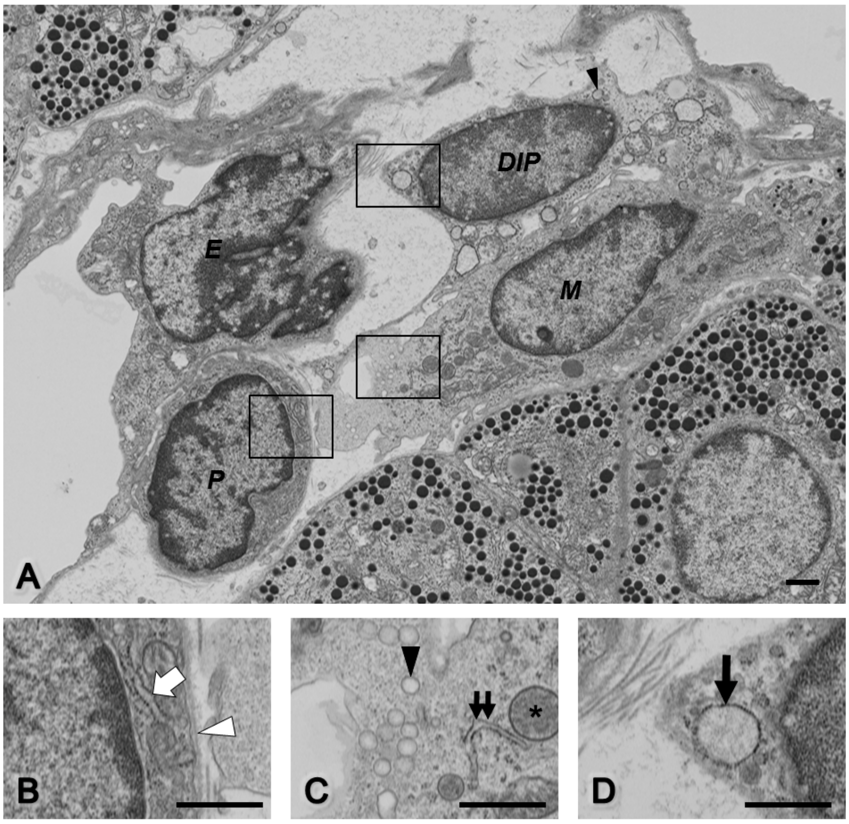

Figure S2. Fine structures of three types of perivascular cells, i.e., pericytes (*P*), macrophages (*M*), and novel desmin-immunopositive perivascular cells (*DIP*), in the normal rat anterior pituitary gland (**A**–**D**). Only pericytes are covered by the basement membrane (white arrowhead in **B**). Flat cisternae of rERs are visible in pericytes (white arrow in **B**) and macrophages (double arrows in **C**), while novel DIP cells have dilated rERs (black arrow in **D**). Surface of small vesicles (black arrowhead in **C**) and primary lysosomes (asterisk in **C**) in macrophages do not show ribosome lining when compared to the globular rERs in novel DIP cells. Note close contact between novel DIP cells and macrophages. Bars 1 μm (**A**), 0.5 μm (**B**–**D**)

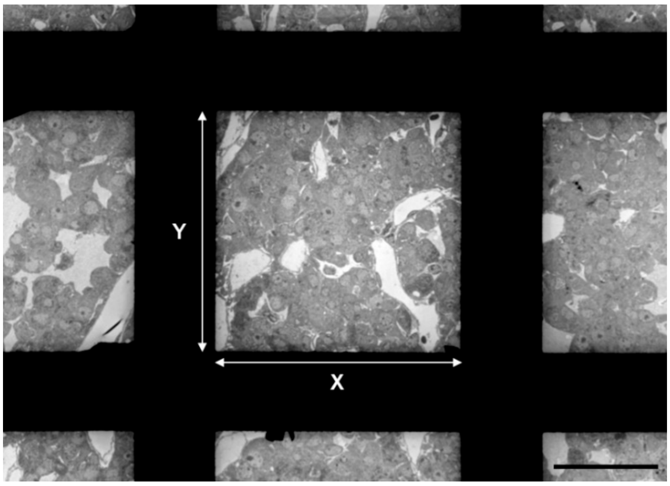

Figure S3. Transmission electron microscopy of the counting area in one square of the 150-mesh square copper grid. X-axis and Y-axis are 118 and 116 μm, respectively. The total counting area per specimen is 36 squares in one grid. Bar 50 μm

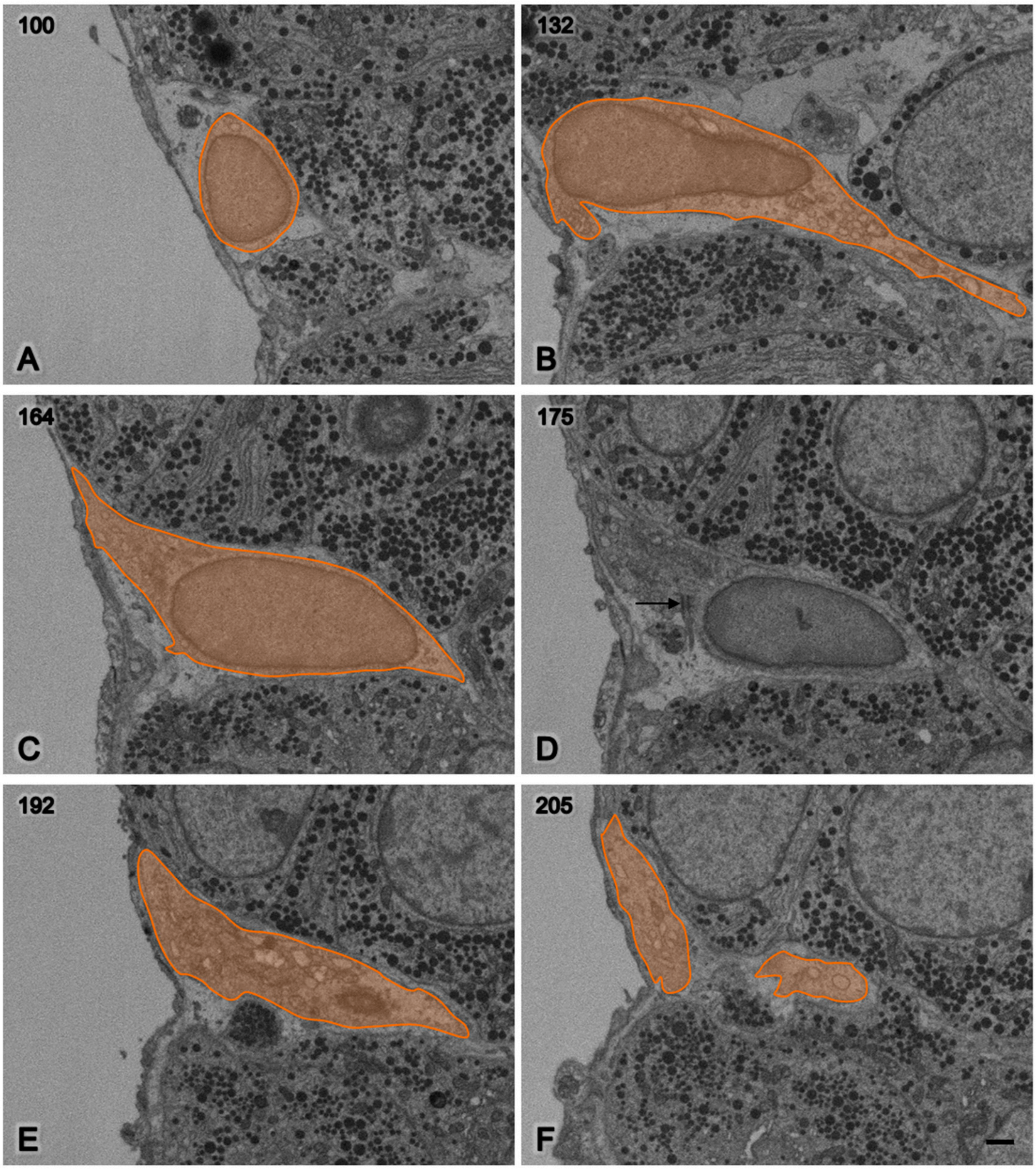

Figure S4. Focused ion beam scanning electron microscopic tomography of a novel desmin-immunopositive perivascular cell in the rat anterior pituitary gland (**A**–**F**). Note a single cilium (arrow in **D**). Bar 1 μm

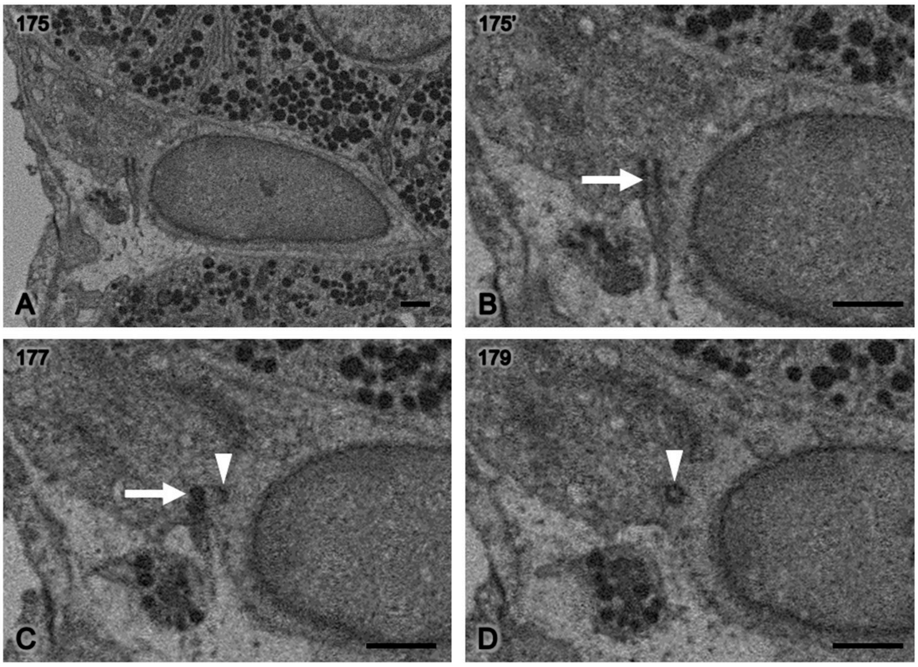

Figure S5. A single cilium of the novel desmin-immunopositive perivascular cell is clearly observed from slide numbers 175 (**A**, **B**: higher magnification of **A**) to 179 (**C**–**D**). Note a mother centriole (arrows in **B** and **C**) and a daughter centriole (arrowheads in **C** and **D**) of centrosomes. Bars 1 μm

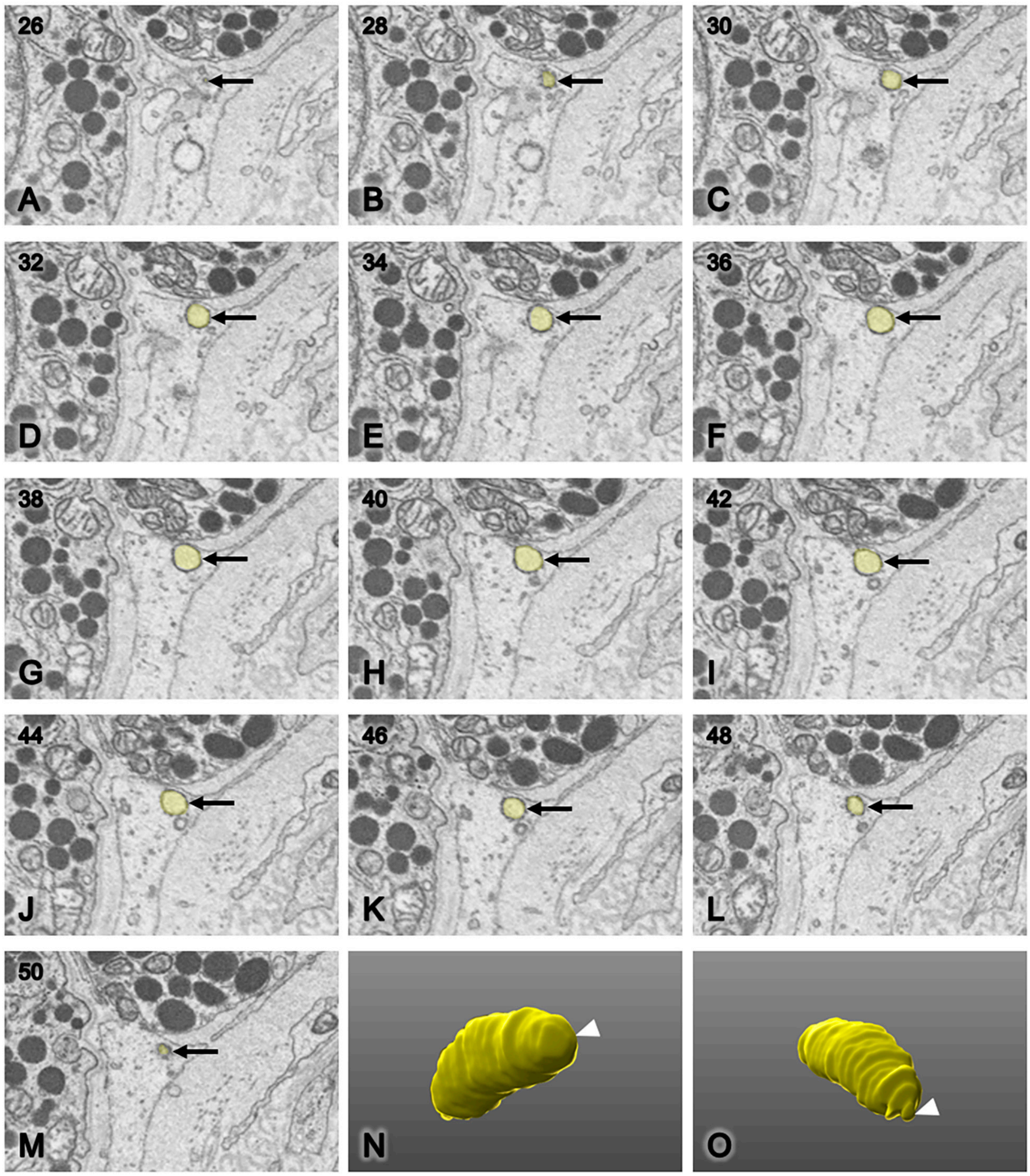

Figure S6. Segmentation of an isolated tubular rER in the cell process of the novel DIP cells (arrows in **A**–**M**) and its three-dimensional reconstruction in the anterior (**N**) and posterior (**O**) views. White arrowheads indicate two blind ends of this rER body. Note that magnification is 6,000×, image pixel size on the XY plane is 9.3042 nm, and resolution on the Z plane is 200 nm.

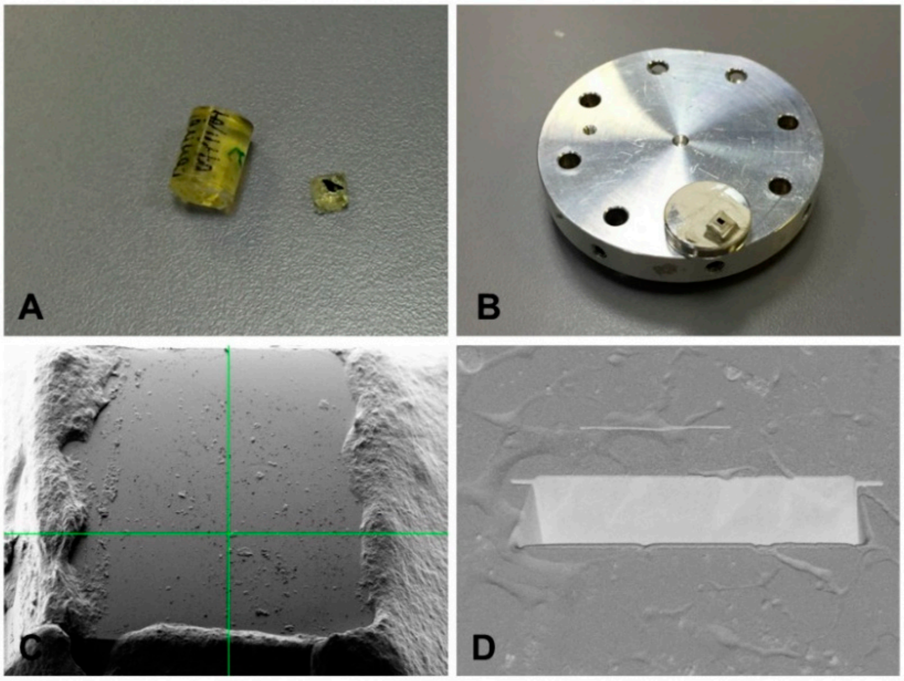

Figure S7. Conventional epoxy resin block (**A**), resin piece containing tissue on the specimen holder (**B**), metal coating on the resin block (**C**), and milling-scanning area using focused ion beam electron microscopy on the resin block (**D**).
